# The Relation between Reading Skills and Eye Movement Patterns in Adolescent Readers: Evidence from a Regular Orthography

**DOI:** 10.1371/journal.pone.0145934

**Published:** 2016-01-04

**Authors:** Magdalena Krieber, Katrin D. Bartl-Pokorny, Florian B. Pokorny, Christa Einspieler, Andrea Langmann, Christof Körner, Terje Falck-Ytter, Peter B. Marschik

**Affiliations:** 1 Research Unit iDN–interdisciplinary Developmental Neuroscience, Institute of Physiology, Center for Physiological Medicine, Medical University of Graz, Graz, Austria; 2 Machine Intelligence & Signal Processing Group, Technische Universität München, Munich, Germany; 3 BEE-PRI: Brain, Ears & Eyes–Pattern Recognition Initiative, BioTechMed-Graz, Graz, Austria; 4 Department of Strabism, Pediatric Ophthalmology and Rehabilitation of Visually Impaired, Medical University of Graz, Graz, Austria; 5 Cognitive Psychology & Neuroscience, Department of Psychology, University of Graz, Graz, Austria; 6 Center of Neurodevelopmental Disorders (KIND), Department of Women’s and Children’s Health, Karolinska Institutet, Stockholm, Sweden; 7 Department of Psychology, Uppsala University, Uppsala, Sweden; The University of Nottingham, UNITED KINGDOM

## Abstract

Over the past decades, the relation between reading skills and eye movement behavior has been well documented in English-speaking cohorts. As English and German differ substantially with regard to orthographic complexity (i.e. grapheme-phoneme correspondence), we aimed to delineate specific characteristics of how reading speed and reading comprehension interact with eye movements in typically developing German-speaking (Austrian) adolescents. Eye movements of 22 participants (14 females; mean age = 13;6 years;months) were tracked while they were performing three tasks, namely silently reading words, texts, and pseudowords. Their reading skills were determined by means of a standardized German reading speed and reading comprehension assessment (*Lesegeschwindigkeits- und -verständnistest für Klassen 6−12*). We found that (a) reading skills were associated with various eye movement parameters in each of the three reading tasks; (b) better reading skills were associated with an increased efficiency of eye movements, but were primarily linked to spatial reading parameters, such as the number of fixations per word, the total number of saccades and saccadic amplitudes; (c) reading speed was a more reliable predictor for eye movement parameters than reading comprehension; (d) eye movements were highly correlated across reading tasks, which indicates consistent reading performances. Contrary to findings in English-speaking cohorts, the reading skills neither consistently correlated with temporal eye movement parameters nor with the number or percentage of regressions made while performing any of the three reading tasks. These results indicate that, although reading skills are associated with eye movement patterns irrespective of language, the temporal and spatial characteristics of this association may vary with orthographic consistency.

## Introduction

Fluent and accurate reading, as well as reading comprehension, are key abilities for academic achievement. The process of reading has been studied extensively in competent readers, but also in individuals with reading disabilities (for reviews, see [1,2]). In addition to the assessment of reading speed, accuracy and/or comprehension, the analysis of eye movements has become increasingly widespread in reading research. Due to the association between eye movements and attention (for a review, see [3]), eye tracking serves as a tool for investigating the real-time course of cognitive processes during reading. A number of researchers associated differences in reading skills with these cognitive processes. For example, fast readers (a) were found to produce fewer fixations, fixations of shorter duration, larger saccades, and fewer regressions (e.g., [4,5]), and (b) showed larger perceptual spans (or letter identity spans) when reading sentences and texts (e.g., [4,6]). Highly skilled readers showed shorter fixation durations and fewer regressions than average readers (e.g., [7,8]).

Research on developmental aspects of eye movements during reading has yielded the following characteristics: compared with adult readers, children showed (a) more frequent saccades and regressions, smaller saccadic amplitudes, prolonged fixation durations and total reading task durations, as well as higher amplitudes of disconjugacy [9]; and (b) an even more pronounced word-length effect with respect to temporal and spatial eye movement parameters (i.e. gaze duration and refixation probability) [10]. In addition, (c) the perceptual span (or letter identity span; visual span) increased with age [6,11,12]; and (d) viewing duration, first fixation duration and refixation time decreased (in line with fixation probability and refixation frequency), while the number of interword regressions increased during the first five school grades, with the largest step taken between the 1^st^ and 2^nd^ grades [13]. However, some typical characteristics of adult eye movement patterns were already observed in children at the earliest stages of reading development, including the same landing positions of saccades and similar refixation likelihood patterns following suboptimal landing positions [10,14,15]. Summarizing current knowledge about reading development, Blythe and Joseph [16] recently concluded that the eye movement behavior during reading approaches adult characteristics around the age of 11. By comparing slow and fast readers of different ages, Häikiö and colleagues [6] showed that some of their findings (e.g., characteristics of the letter identity span) were more likely attributable to reading skills than to age.

A great deal of studies have dealt with the influence of reading skills on eye movements in orthographically inconsistent (i.e. irregular with regard to the grapheme-phoneme correspondence) languages, such as English or French (e.g., [4,5,7–12,14,15]). Ziegler and Goswami [17] elaborated on the grapheme-phoneme correspondence and its impact on reading development. One critical difference between regular and irregular orthographies is the size of units readers have to decode to ascertain the correct pronunciation of graphemes. As compared with regular orthographies like German or Italian, graphemes may be pronounced differently in more irregular orthographies. Therefore, readers of orthographically inconsistent languages cannot rely on grapheme-phoneme conversion but have to decode linguistic units of bigger sizes (e.g., syllables or even whole words) [17].

Whether or not the influence of reading skills on eye movements is language-specific is still not fully understood. Rau et al. [18] recently found cross-linguistic differences in the eye movements of English- and German-speaking readers, notably in children and adults. They attributed their findings to language-immanent characteristics such as the use of compounds.

To date, only few studies have focused on developmental aspects of eye movements in German readers (e.g., [13,19]). Most studies investigating the influence of reading skills on eye movements in orthographically consistent languages such as German or Italian have focused on a comparison of dyslexic and normal readers (e.g., [20–29]). Examining the eye movements of adolescent readers, Hutzler and Wimmer [20] found dyslexics to show more frequent fixations and longer fixation durations. Regressions were generally infrequent, with a slightly higher proportion of regressions in the group of dyslexics. Trauzettel-Klosinski and colleagues [21] found dyslexic children to produce a larger total number of saccades and regressions. The percentage of regressions was only slightly increased and fixation duration did not differ from typical readers. Similar results were reported for adult German-speaking dyslexics [22]. These findings are in contrast with those on English-speaking dyslexics, whose eye movement patterns typically showed prolonged fixation durations and substantially higher numbers of regressions (for reviews, see [1,30]).

As mentioned above, a large number of studies carried out in regular orthographies compared eye movements of dyslexic and typical readers. Research on the relation between reading skills and eye movements in typical (i.e. non-pathological) readers of languages with regular orthographies is scarce. As comparisons between dyslexics and typical readers do not account for the variability in typical reading skills, there is little knowledge on potential differences in the influence of typical reading skills on reading strategies in regular and irregular orthographies. With our study we aimed to determine how reading skills (speed and comprehension) were related to eye movement patterns in typically developing German-speaking (Austrian) adolescents. We posed the following questions: (a) How does the range of normal reading skills (speed and comprehension) relate to eye movements? (b) To what extent is the relation between reading skills and eye movement patterns task-specific? (c) Are individual eye movement patterns consistent across different reading tasks?

## Methods

### Participants

Our sample consisted of 23 typically developing, monolingual German-speaking adolescents (14 females), aged 13;6 ± 0;2 (years;months) who volunteered to participate in the study. All of them also participated in a comprehensive longitudinal study on typical neuromotor, cognitive, and speech-language development (e.g., [31–33]). They were born within a 3-week-interval in August/September 1998 after an uneventful pregnancy at the Department of Obstetrics and Gynecology at the University Hospital of Graz, Austria. Neurological examinations and developmental assessments during their first year of life yielded normal scores.

The study adhered to the principles of the Declaration of Helsinki and was approved by the Institutional Review Board of the Medical University of Graz, Austria. After the experimental procedure was explained to them, all 23 adolescents assented to participate. Parents provided written informed consent for participation and the publication of results.

### Ophthalmologic and orthoptic examinations

To check for visual impairments, we advised our participants to attend ophthalmologic and orthoptic examinations at the Department of Ophthalmology, Medical University of Graz, Austria. From 15 participants who underwent the ophthalmologic and orthoptic examinations, 14 participants had normal or corrected-to-normal vision. One participant (participant 4, see Table 1) showed esotropia and was therefore excluded from analysis, resulting in a final sample of N = 22.

**Table 1:** Sample characteristics.

		LGVT [34] reading skills (raw scores)	
*Participant*	*Gender*	*Reading speed*	*Reading comprehension*
1	m	493	6
2	f	649	4
3	f	493	4
4	m	na	na
5	m	491	9
6	f	415	10
7	m	567	13
8	f	621	10
9	m	499	6
10	f	594	16
11	f	411	4
12	f	684	15
13	m	491	9
14	m	504	8
15	f	462	6
16	f	630	13
17	f	727	15
18	m	702	6
19	f	895	21
20	f	907	18
21	f	610	13
22	f	434	12
23	m	983	26
	**9m/14f**	**Mean = 602.8**	**Mean = 11.1**

The sample’s gender distribution is given in the middle column; the section “LGVT reading skills (raw scores)” shows each participant’s raw score for reading speed and reading comprehension. Key: LGVT = Lesegeschwindigkeits- und -verständnistest für Klassen 6−12 [Reading Speed and Reading Comprehension Test for Grades 6−12] [34]; f = female; m = male; na = not applicable

### Assessment of reading skills

As a first step, we assessed the participants’ reading skills using the *Lesegeschwindigkeits- und -verständnistest für Klassen 6−12* (LGVT [Reading Speed and Reading Comprehension Test for Grades 6−12]) [34], a standardized test for evaluating reading competence (i.e. reading speed and reading comprehension) in German-speaking adolescents. The LGVT consists of a text with 1727 words. At 23 predefined positions in this text participants have to pick one of three words that they think fits into the given context. Reading time is restricted to 4 minutes. The manual of the LGVT includes a standardized instruction, which adds up to a total test duration of approximately 10 minutes. The reading speed score corresponds to the total number of words read within 4 minutes; as for the reading comprehension score, each correct answer scores 2 points while 1 point is deducted for each incorrect answer. The LGVT shows a high consistency for both subscales, with retest-reliability coefficients of *r* = .84 and *r* = .87 [34].

### Assessment of eye movements

To determine the dominant eye we asked the participants to look through a kaleidoscope.

We recorded binocular eye movements using a video-based corneal reflection eye tracker (iView X Hi-Speed, Sensomotoric Instruments; http://www.smivision.com) with a sampling rate of 500 Hz (tracking resolution: <0.01 degrees of visual angle [dva]).

Eye movements were assessed during the following reading tasks: (a) 3 German text reading tasks (A1, A2, A3); (b) 1 German word reading task (B); and (c) 3 pseudoword reading tasks (C1, C2, C3). The first text (A1) contained 150 words and was taken from a school book for eighth-graders ([35], pages 7–8). To achieve a good comparability of results, the second (A2, 29 words) and third texts (A3, 31 words) were taken from Hutzler and Wimmer ([20], page 241), who had used these texts to compare reading strategies of dyslexic seventh-graders with age-matched controls. B (135 words) and C1 (111 pseudowords) were taken from the *Salzburger Lese- und Rechtschreibtest II* (SLRT II [Salzburg Reading and Spelling Test II]; Form-A lists) [36], a standardized inventory for measuring reading fluency and spelling. For the same reasons as presented for text reading materials, C2 (31 pseudowords) and C3 (29 pseudowords) were also taken from Hutzler and Wimmer ([20], page 241).

### Procedure

All experimental assessments were carried out at the BRAIN*tegrity* lab at the Institute of Physiology, Medical University of Graz, Austria, between December 2011 and June 2012. During the experimental procedure the lab was dimly lit. The chair and table were individually adjusted for each participant. A chin and a forehead rest were used to limit head movements. Stimuli were presented on a 22-inch monitor with a resolution of 1680 x 1050 and a refresh rate of 60 Hz. The viewing distance was set at 90 cm, resulting in a maximum screen width of 29.5 dva and a maximum screen height of 18.7 dva. The monitor’s centre was positioned 10 cm below the eyes and the monitor’s tilt angle was set at 6.4°.

Each participant underwent a 13-point calibration procedure, which was repeated until they achieved an average gaze position error below 0.4 dva. The participants were instructed to read silently, comprehensively and carefully. To ensure careful reading, they were informed that the investigator followed their eye movements on a separate monitor.

Reading materials were displayed in black Courier New font on a white screen. Letters subtended a width of approximately 0.5 dva. Each page consisted of up to four double-spaced text lines with an average width of 18.3 dva. Words and pseudowords were separated by a comma and a blank. For text reading, the page breaks were adjusted according to sentence endings. Each reading task was presented separately, numbering 2 to 11 pages. To indicate that they had finished reading a page, the participants were instructed to fixate the lower right corner of the monitor. As soon as a stable corner fixation was given, the instruction “Bitte blinzeln!” [Please blink!] appeared.

### Analysis of eye movements

We only analyzed the movements of the dominant eye, using SMI BeGaze (Sensomotoric Instruments; http://www.smivision.com), and considered the following parameters: (a) average fixation duration (in ms); (b) number of fixations per word; (c) first fixation duration (i.e. duration of the initial fixation on a word; in ms); (d) number of saccades; (e) saccadic amplitude (in dva); (f) number of regressions; (g) percentage of regressions; and (h) gaze duration (i.e. summed up fixation duration on a word before moving to another word either in or against reading direction; in ms). In addition to eye movement parameters we assessed the total reading time (in ms) for each task. Note that the number and percentage of regressions include both intra- and interword regressions.

For saccade detection, the peak velocity threshold was set at 40°/s and the minimum saccade duration was 22 ms. The minimum duration for fixation detection was set at 50 ms. The word reading data (B) of participant 13 and the pseudoword reading data (C2 and C3) of participant 8 were partly missing because of technical problems (loss of track of the participants’ eyes). Since incomplete data sets were excluded from the analysis, word reading and pseudoword reading were thus computed for 21 participants.

In line with Hutzler and Wimmer [20] we pooled the data from the two text reading tasks A2 and A3 (A2+A3; 60 words) as well as those from the two pseudoword reading tasks C2 and C3 (C2+C3; 60 words). We calculated Cronbach’s alpha to evaluate the internal consistency of reading times and eye movement parameters for text reading tasks A1 and A2+A3, and pseudoword reading tasks C1 and C2+C3. Total reading times were highly correlated in case of C1 and C2+C3, and acceptably correlated in case of A1 and A2+A3 ([37], page 231; Table 2). For the total number of saccades and regressions, internal consistencies were acceptable to good for both, A1 and A2+A3, as well as C1 and C2+C3. All other internal consistencies were good to excellent and therefore suited to summarize the results for word and pseudoword reading. ([37], page 231)

**Table 2:** Cronbach’s alpha.

	Text reading (A1, A2+A3)	Pseudoword reading (C1, C2+C3)
**Total reading time**	.72	.92
**First fixation duration**	.91	.88
**Average fixation duration**	.90	.95
**Gaze duration**	.92	.96
**Number of fixations per word**	.93	.94
**Total number of saccades**	.79	.85
**Total number of regressions**	.72	.85
**Percentage of regressions**	.93	.86
**Saccadic amplitude**	.96	.90

Cronbach’s alpha for total reading times and eight eye movement parameters across three reading tasks for text reading (A1, A2+A3) and pseudoword reading (C1, C2+C3).

### Statistics

Statistical analyses were performed using IBM SPSS Statistics 22 (SPSS Inc., Chicago, IL), with *p* < .05 indicating a statistically significant effect. To assess the predictability of eye movements, data were entered into multiple linear regression analyses. Correlations were assessed using Pearson’s product-moment correlation. For correlation and regression analyses including the variable percentage of regressions, participant 10 was excluded due to a highly increased mean (more than 3 standard deviations above the sample’s mean).

## Results

We will first report results on reading skills, i.e. results of the LGVT assessments. We shall then provide results on the relation between LGVT reading skills and eye movement parameters for word reading, text reading, and pseudoword reading. In a third step, we will focus on the consistency of eye movement parameters across reading tasks.

### Reading skills

Participants achieved a mean of 602.8 words per 4 minutes (*SD* = 161.7; *Range* = 411–983) in the reading speed assessment, and a mean of 11.1 points (*SD* = 5.8; *Range* = 4–26) in the reading comprehension assessment based on LGVT.

There was a positive correlation of *r* = .78 (*p* < .001) between reading speed and reading comprehension: individuals who scored higher in the reading speed assessment also performed better in the reading comprehension assessment.

### Relation between LGVT reading skills and total reading times as well as eye movement parameters

Table 3 shows the means and standard deviations for total reading times and the eight eye movement parameters for each of the three reading tasks (i.e. word reading, text reading, pseudoword reading). Note that cumulative variables such as total reading time, the number of fixations per word, the total number of saccades and the total number of regressions are not comparable across reading tasks because of differences in the total number of words and word lengths.

**Table 3:** Descriptive characteristics of total reading times and eye movement parameters for three reading tasks.

	Word reading (135 words)		Text reading (210 words)		Pseudoword reading (171 pseudowords)	
	*Mean*	*SD*	*Mean*	*SD*	*Mean*	*SD*
**Total reading time (ms)**	77177	17963	77101	16877	141338	42688
**First fixation duration (ms)**	283	43	210	22	321	54
**Average fixation duration (ms)**	261	37	204	22	289	41
**Gaze duration (ms)**	478	122	308	61	700	227
**Number of fixations per word**	1.96	.41	1.46	.27	2.58	.69
**Total number of saccades**	163.1	31.8	197.6	44.4	244.3	48.2
**Total number of regressions**	32.7	14.0	40.1	16.8	49.4	18.9
**Percentage of regressions**	15.8	6.0	15.6	8.4	16.1	5.0
**Mean saccadic amplitude (dva)**	3.2	.53	3.4	.70	2.6	.52

Means and standard deviations (*SD*) for total reading times, and eight eye movement parameters for word reading, text reading and pseudoword reading. Key: dva = degrees of visual angle

For each of the parameters assessed, we performed a multiple linear regression with LGVT reading speed and LGVT reading comprehension as predictor variables. Due to the high correlation between the predictors, beta weights may not validly indicate the contribution of each predictor. Hence, to estimate how shared variance of the predictor variables may have affected the estimation of beta weights, we decided to additionally report the zero-order correlation between each predictor and the criterion.

#### Word reading

The results of the multiple linear regressions for word reading are given in the left column of Table 4. LGVT reading skills significantly predicted the number of fixations per word (*F* = 4.54, *p* < .05) and the mean saccadic amplitude (*F* = 10.09, *p* < .01), accounting for 34% and 53% of variance in the respective variables. Additionally, there was a statistical trend indicating an influence of LGVT reading skills on gaze duration and the total number of saccades, but the equations failed to reach significance (*F* = 3.20, *p* = .065 and *F* = 3.39, *p* = .056). However, LGVT reading skills did not predict total reading time, first or average fixation duration, or any of the regression parameters (total number of regressions and percentage of regressions; all *F*s<2.50, *p*s>.05). These results suggest that, when reading word lists, better readers make larger saccades, fixate less frequently and tend to spend less time on fixating words during first-pass reading than less skilled readers.

**Table 4:** Results of the multiple linear regression analyses.

	Word reading					Text reading					Pseudoword reading				
		*Reading speed*		*Reading comprehension*			*Reading speed*		*Reading comprehension*			*Reading speed*		*Reading comprehension*	
	R^2^	β	r	β	r	R^2^	β	r	β	r	R^2^	β	r	β	r
**Total reading time**	.216	-.537	-.461	.098	-.319	.492**	-.699*	-.701	-.003	-.546	.246^+^	-.433	-.494	-.078	-.415
**First fixation duration**	.202	-.650^+^	-.115	.689^+^	.184	.330*	-.834*	-.511	.415	-.233	.105	-.496	-.265	.297	-.089
**Average fixation duration**	.141	-.471	-.008	.596	.231	.222^+^	-.697*	-.410	.370	-.172	.091	-.480	-.211	.346	-.028
**Gaze duration**	.262^+^	-.708*	-.477	.298	-.252	.535***	-.917**	-.712	.264	-.449	.306*	-.540	-.553	-.017	-.438
**Number of fixations per word**	.335*	-.412	-.565	-.198	-.518	.528***	-.581*	-.718	-.176	-.628	.260^+^	-.281	-.483	-.259	-.478
**Total number of saccades**	.274^+^	-.391	-.514	-.158	-.461	.531***	-.500^+^	-.709	-.268	-.657	.165	-.303	-.398	-.123	-.359
**Total number of regressions**	.074	-.303	-.271	.040	-.194	.015	-.185	-.041	.185	.041	.035	-.174	-.186	-.016	-.152
**Percentage of regressions**^1^	.040	.254	-.007	-.330	-.128	.087	.429	.267	-.205	.136	.068	.364	.024	-.428	-.138
**Mean saccadic amplitude**	.529**	.755**	.727	-.036	.550	.653***	.735**	.806	.092	.663	.318*	.534	.563	.038	.454

Squared multiple correlations (R^2^), beta weights (β) and zero-order correlation coefficients (r) for total reading times, and eight eye movement parameters for word reading, text reading and pseudoword reading.

Key: ^1^ = Participant 10 was excluded due to a highly increased mean (more than 3 standard deviations above the sample’s mean).

***p < .001

**p < .01

*p < .05

^+^p < .10

As indicated by the beta weights, LGVT reading speed was the more important predictor in the equation for mean saccadic amplitude. On the other hand, the significant prediction of the number of fixations per word resulted from the combined predictive power of both reading skill variables, as both predictors failed to reach significance. In case of the marginally significant models for gaze duration and the total number of saccades, we observed a similar pattern: regarding gaze duration, beta weights indicated a unique contribution of LGVT reading speed, while the explained variance in the total number of saccades resulted from a linear combination of both predictors. Beta weights indicated that LGVT reading comprehension could not explain unique variance in any of the four (marginally) significant models. However, zero-order correlation coefficients suggested that–at least for the explanation of saccadic amplitudes–LGVT reading comprehension could also be a useful predictor, but was not credited in the equation, as it did not independently contribute to R^2^ in the respective variable.

#### Text reading

The middle column of Table 4 shows the results of the multiple linear regressions for text reading. In contrast to word reading, in case of text reading LGVT reading skills significantly predicted a larger number of eye movement parameters, with regression equations accounting for 33% to 65% of variance in the dependent variables. Better reading skills predicted a decrease in total reading time (*F* = 9.19, *p* < .01), first fixation duration (*F* = 4.68, *p* < .05), gaze duration (*F* = 10.93, *p* < .001), the number of fixations per word (*F* = 10.61, *p* < .001), and the total number of saccades (*F* = 10.76, *p* < .001), as well as an increase in the saccadic amplitude (*F* = 17.91, *p* < .001). In addition, we found a marginally significant equation for average fixation duration (*F* = 2.71, *p* = .092). As for word reading, LGVT reading skills neither predicted the total number of regressions nor the percentage of regressions (both *F*s<1, *p*s>.05).

As indicated by the beta weights, LGVT reading speed was the more important predictor than reading comprehension in all of the significant equations for text reading. Beta weights for reading comprehension were consistently lower and even near zero for total reading time and saccadic amplitude. As for word reading, correlation coefficients suggested that LGVT reading comprehension shared a considerable amount of variance with R^2^, but did not additionally contribute to the variance explained by LGVT reading speed.

Compared to word reading, LGVT reading skills were found to interact with a larger number of eye movement parameters, especially with regard to temporal parameters. Besides an only marginally significant equation for average fixation duration, higher LGVT reading skills were consistently related to an increased efficiency in the majority of temporal and spatial parameters.

#### Pseudoword reading

The results of the multiple linear regressions for pseudoword reading are presented in the right column of Table 4. The regression equation revealed significant results for gaze duration (*F* = 3.97, *p* < .05) and saccadic amplitudes (*F* = 4.20, *p* < .05), with LGVT reading speed and LGVT reading comprehension explaining about 31% and 32% of the dependent variables’ variances. Again, we found some statistical trends indicating an effect of LGVT reading skills: the models for total reading time and the number of fixations per word just failed to reach significance (*F* = 2.94, *p* = .079 and *F* = 3.16, *p* = .067). Similar to the results for word reading, these results indicated that better readers make larger saccades, fixate less frequently and tend to spend less time on fixating words during first-pass reading than less skilled readers.

As for word and text reading, in most of the (marginally) significant equations, beta weights were considerably higher for reading speed than for reading comprehension. However, all beta weights failed to reach significance and correlation coefficients showed that both LGVT assessments could have accounted for a substantial amount of variance in the dependent variables.

### Correlations between total reading times and eye movements across reading tasks

We assessed the coherence of the participants’ reading performances across reading tasks. We used Pearson’s product-moment correlation coefficient to correlate the participants’ individual performances on three pairs of reading tasks (word reading × text reading; text reading × pseudoword reading; and word reading × pseudoword reading). Table 5 shows that, except for the percentage of regressions, which was only marginally significant between text reading and pseudoword reading (*p* = .094), all eye movement parameters were strongly associated across reading tasks (*r*s ranging from .55 to .89). And except for the percentage of regressions, correlations for spatial parameters (i.e. number of fixations per word, total number of saccades and regressions, and saccadic amplitude) were slightly stronger than for temporal parameters (i.e. first fixation duration, average fixation duration, and gaze duration). For all eye movement parameters, correlation coefficients between text and pseudoword reading tended to be weaker than between the other two pairs of reading tasks.

**Table 5:** Correlations between total reading times and eye movement parameters across reading tasks.

	Word reading × text reading	Text reading × pseudoword reading	Word reading × pseudoword reading
**Total reading time**	.84***	.66***	.74***
**First fixation duration**	.67***	.58**	.75***
**Average fixation duration**	.70***	.68***	.70***
**Gaze duration**	.81***	.55**	.70***
**Number of fixations per word**	.89***	.68***	.74***
**Total number of saccades**	.89***	.67**	.81***
**Total number of regressions**	.69***	.66**	.88***
**Percentage of regressions**^1^	.70***	.38	.68**
**Mean saccadic amplitude**	.86***	.70***	.87***

Pearson’s product-moment correlation coefficients between total reading times, and eight eye movement parameters for three pairs of reading tasks (i.e. word reading × text reading; text reading × pseudoword reading; and word reading × pseudoword reading).

Key: ^1^ = Participant 10 was excluded due to a highly increased mean (more than 3 standard deviations above the sample’s mean).

***p < .001

**p < .01; *p < .05

## Discussion

The relation between reading competence and eye movement patterns has to a great extent been studied in irregular orthographies; many of these studies compared dyslexic with skilled readers. Assessing eye movements in a sample of typical readers in a regular orthography, we found that (a1) better reading skills (speed and comprehension) were associated with more efficient eye movements, primarily reflected by reduced spatial parameters. (a2) Compared to reading comprehension, reading speed tended to be more reliable as a predictor for eye movement parameters, although both of them shared a considerable amount of variance with R^2^ in the majority of significant equations. (a3) The few temporal eye movement parameters (i.e. gaze duration and–for text reading–first and average fixation duration) that were influenced by reading skills were primarily related to reading speed, while reading comprehension only shared a minor amount of variance with R^2^. Regarding the task-specificity of eye movement parameters, our results revealed (b) a relation with reading skills for different reading tasks (i.e. word, text and pseudoword reading). The most reliable predictions were observed for text reading. In addition, we found (c) high intraindividual consistencies between reading tasks.

Similar to studies comparing German- or Italian-speaking adolescents with and without dyslexia (e.g., [20,24,25]), we found German reading skills in typically developing individuals to be consistently related to parameters corresponding to (i) the amount and (ii) the length of forward movements of the eyes (number of fixations per word, total number of saccades, saccadic amplitude). Gaze duration was also reliably predicted by LGVT reading skills in the text and pseudoword reading tasks, and marginally in the word reading task. In line with the only small effect reported in the aforementioned studies (e.g., [20,24,25]), regressive eye movements (total number of regressions, percentage of regressions) did not vary with reading skills in either of the reading tasks. Prolonged first and/or average fixation durations for less skilled readers, as reported for English-speaking cohorts (e.g., [4,7,38]) and by Hutzler and Wimmer [20], could only be observed for text reading. Although our results showed the same trend as previously reported for German (e.g., [20–22]), we assume that fixation duration only accounts for a small amount of variance in typical German readers. Instead, the extra time needed to process written information during first-pass reading appears to be mainly caused by the interaction of reading skills and spatial eye movement parameters. This is in line with De Luca and colleagues [25], who found that Italian dyslexic readers fixate longer compared to typical readers, but differences with regard to spatial parameters were more pronounced.

Our findings further support the assumption that, in regular orthographies, better reading skills may lead to a decrease in the number of eye movements necessary to process written information [20]. Additional evidence to support this assumption may lie in the high consistency of eye movement parameters across reading tasks in spite of the fact that task demands varied greatly (e.g., word reading vs. pseudoword reading). Studies on reading conducted in languages with irregular orthographies, such as English, have shown temporal and regressive eye movement parameters to be consistently related to reading skills (e.g., [4,7]).

When comparing different studies, one has to be aware that some of these focused on reading speed performance (e.g., [4,6]), while others chose comprehension or a combination of different reading skills to determine reading competence (e.g., [7,8,38]).

## Limitations

One limitation of our study is the relatively small sample size. All participants were part of a 13-year longitudinal study (e.g., [31–33]) with a drop-out rate of 50%. Therefore, this study was predominantly exploratory and has a suggestive character. To increase the validity of its results, evidence from a larger sample is required. A second limitation lies in the norms available to determine the participants’ percentile ranks for reading speed and reading comprehension. The LGVT [34] was normalized in Germany; because the German and Austrian school systems differ to some extent, percentile ranks for different school types may not be optimal for an Austrian sample.

## Conclusions

Our study adds to the knowledge on the relation between reading skills and eye movement behavior in different reading tasks. Assessing the predictive power of reading speed and reading comprehension for eye movement behavior in typical adolescent German readers representing the broad spectrum of normality, our results fit in the existing body of literature. A strength of our study is the good validity of the reading material chosen, as illustrated by the high consistency of eye movements across the reading tasks. We emphasize the surprisingly high variability of reading skills in our small sample of typically developing adolescents.

Our data suggest (i) that both reading speed and reading comprehension interacted with eye movements during three different reading tasks (word, text and pseudoword reading), though reading speed was a more reliable predictor for eye movements than reading comprehension, and (ii) that German reading skills were more closely related to spatial than to temporal eye movement parameters. In line with previous work by Hutzler and Wimmer [20], the present study indicates that in German, a language with regular orthography, the influence of reading skills on eye movement patterns may be different from that reported for irregular orthographies, such as English (e.g., [4,7]).

Further research is needed to reveal potential language-specific influences of reading skills on eye movements in normal readers of different ages. Future studies may (a) consider reading skills as a continuous feature, and (b) assess different aspects of literacy competence.
